# Glances at the Hospitals

**Published:** 1901-02-02

**Authors:** 


### GLANCES AT THE HOSPITALS.

### ADDENBROOKE'S HOSPITAL, CAMBRIDGE.

THIS hospital was founded in the year 1719 by John Addenbrooke, M.D., but the hospital was not opened for the reception of patients until 1766. In 1814 it was enlarged with money left by Mr. John Bowtell; but in 1864 it was almost entirely rebuilt, and greatly enlarged, and in 1878 £3,590 was spent in further additions. It now contains 158 beds. Excluding sums invested the income for 1899 was £6,454, and £500, being the proceeds of sale of land, was added to the current account. The expenditure reached £7,744. There were 1,379 patients treated in the hospital, and 6,444 out-patients. The daily average number resident was 98, and the average stay in the hospital was 26 days. The report is unusually deficient in such information as the medical or general reader would care about. Neither medical nor surgical tables are given—an omission which seems the more extraordinary as a medical school is attached to the hospital.

### THE SUNDERLAND INFIRMARY.

THE income of this hospital for 1899 amounted to £10,682, including bequests of £600. Of the total sum we notice that the annual subscriptions amounted to £1,784, and the workpeople's contributions to £6,116; the latter being £540 higher than they were in 1898, a feature which the Committee may well describe as "gratifying." The total expenditure was £10,860; but £600 of this was transferred to the Convalescent Home at Harrogate, £100 to the Nurses' Pension Fund; £366 on drainage and £1,066 on the laundry. A sum of £300 is about to be spent on the operating theatre, and in this connection the Sunderland Engineering Company have set a good example by providing the electric lighting installation free of charge. There were 189 patients in the hospital on the 1st of January, and with the admissions made a total of 2,647, showing an increase of 167 over the number for 1898. The out-patients rose from 4,753 in 1898 to 5,107 in 1899, a fact which is not wholly to be desired. The average stay in the hospital was 24 days. The death-rate was 5·44 per cent. of the whole number of cases; but if 39 cases moribund on admission be omitted the death-rate would fall to 3·96 per cent. The cost of maintenance per bed occupied was £46 19s., and the cost per patient was £3 2s. 4d. Payments by patients amounted to £93. The total cost of the out-patients was £476. The hospital contains 210 beds, and the number of the household is 67. The medical cases numbered 1,128, and the surgical 1,519. All of these cases are carefully tabulated and can be followed till their termination in recovery, improvement, or death. There were 26 operations for the radical cure of hernia, and of these 24 were successful and 2 were still under treatment. Abdominal section was performed 47 times (several being for malignant disease), and 25 of these operations were successful; 2 partially so, 1 not successful; 16 died and 3 were under treatment. All the ovariotomies were successful. The total number of operations was 606, and chloroform was given in 594 of these. It will be seen that the report is carefully made out and contains all essential information.

### THE SWANSEA GENERAL HOSPITAL.

THE ordinary income from all sources was £4,761; the ordinary expenditure £4,797; and there is a balance due to the treasurer of £1,391. On turning to the balance-sheet we find that the annual subscriptions and donations amounted to £1,405; and the workpeople's contributions to £1,320; and the committee are not without hope that this sum will be raised to £2,000. The cost per bed occupied was £57 1s., but deducting a reasonable sum for the expenses of the out-patient department, the cost would be reduced to £52 19s. The new operating theatre is now being used. It was the gift of Mr. B. Evans. From the report of the Board of Management we learn that 1,321 in-patients were treated during the year, and that the out-patients numbered 4,636; but otherwise the report comes to us quite innocent of medical tables or details, so we regret we cannot give our readers the usual synopsis.

